# Youth susceptibility to tobacco use in the Gulf Cooperation Council Countries, 2001–2018

**DOI:** 10.1016/j.pmedr.2022.101711

**Published:** 2022-01-30

**Authors:** Sarah S. Monshi, Jingwei Wu, Bradley N. Collins, Jennifer K. Ibrahim

**Affiliations:** a Department of Health Services Administration and Policy, Temple University, Philadelphia, PA, USA; b Department of Health Services Management, Umm Al-Qura University, Mecca, Saudi Arabia; c Department of Epidemiology and Biostatistics, Temple University, Philadelphia, PA, USA; d Department of Social and Behavioral Sciences, Temple University, Philadelphia, PA, USA

## Abstract

The Gulf Cooperation Council (GCC) countries – Bahrain, Kuwait, Oman, Qatar, Saudi Arabia, and the United Arab Emirates (UAE) – ratified the Framework Convention on Tobacco Control (FCTC) in 2006. Yet, GCC countries predict a slight reduction in tobacco use compared to the Eastern Mediterranean region’s significant downward trend. The purpose of this study was to examine changes in self-reported intention to initiating tobacco use (susceptibility) among youth over time in five GCC countries and the relationship between susceptibility and key FCTC provisions. Complex sample logistic regression analyses were conducted using data from the 2001 to 2018 Global Youth Tobacco Survey (n = 349,878 adolescents). Since the ratification of FCTC in GCC countries, susceptibility to initiate tobacco use significantly decreased in Bahrain and Qatar while it increased in UAE (P < 0.001). Exposure to smoking in public places increased the odds of susceptibility to tobacco use in Bahrain (AOR = 1.6, 95% CI = [1.2–2.2), Kuwait (AOR = 1.6, 95% CI = [1.2–2.0]), Qatar (AOR = 1.9, 95% CI = [1.4–2.6]), and UAE (AOR = 2.1, 95% CI = [1.6–2.7]). Susceptibility to tobacco use was significantly associated with exposure to tobacco imagery in media in the UAE (AOR = 1.7, 95% CI = [1.2–2.3]) and with tobacco industry activities like promotion in Bahrain (AOR = 2.8, 95% CI = [1.9–4.2]) and Kuwait (AOR = 2.2, 95% CI = [1.5–3.1]). In conclusion, the impact of FCTC provisions on tobacco use differs across countries. Findings suggested that the implementation of tobacco control policies may independently influence the initiation of tobacco use.

## Highlights

• Analysis of the susceptibility to initiating tobacco use identifies youth at higher risk of initiating tobacco.
• Susceptibility to initiate tobacco use in increased in the United Arab Emirates while decreased in Bahrain and Qatar.
• FCTC may have an independent impact on initiating tobacco use among youth.
• The study helps policymakers identify impactful policies and prioritize resources accordingly.

## 1 Introduction

Tobacco is the cause of more than eight million deaths annually (World Health Organization, 2021). Several countries predict a slight decline in tobacco use, including the Gulf Cooperation Council (GCC) countries (World Health Organization, 2018, Fouad et al., 2021). The GCC is an intergovernmental union of six states: Bahrain, Kuwait, Oman, Qatar, Saudi Arabia, and the United Arab Emirates (UAE). In GCC countries, tobacco was responsible for 16.3% of cancer cases in 2018 (Al-Zalabani, 2020) and approximately US$1.4 trillion in economic costs in 2016 (Koronaiou et al., 2020). While tobacco use in the Eastern Mediterranean is expected to decline from 29.5% in 2000 to 17% by 2025, GCC regionally expects a slight reduction in several countries, for instance, it is expected to decrease from 30.7% in Bahrain in 2000 to 29.8% in 2025 and from 19.9% in 2000 to 19.2% in Saudi Arabia by 2025. Furthermore, the trend of tobacco use in Oman shows an increase from 10.9% in 2000 to 13.5% in 2025. The prevalence of tobacco uses among youth ranges from 11.6% in Kuwait to 1.7% in Oman, with a higher rate among males than females. The smoking trend is increasing among female youth in several GCC countries, including Bahrain, Qatar, Saudi Arabia, and the UAE (World Health Organization, 2019).

### 1.1 Factors associated with susceptibility to tobacco use

Age at initiation is an essential factor in addressing tobacco use because it is linked to subsequent years of tobacco use, nicotine dependence, quit attempts, and tobacco-related diseases (Cantrell et al., 2018). In GCC countries, most current tobacco users start using tobacco before age 18 (Wellman et al., 2016, Nasser et al., 2020). Tobacco use behavior passes through several stages: 1) preparation; 2) initiation; 3) experimentation; 4) regular use 5) addiction (Stern et al., 1987). The point of greatest susceptibility is between the stage of preparation and initiation (Pierce et al., 1996). In this period, an individual starts thinking of initiating tobacco use and perceives positive outcomes from tobacco use (Stern et al., 1987). In addition, physical environment and social contexts, such as homes, schools, and public places, play a fundamental role in creating normative beliefs and expected outcomes related to tobacco use (Aryal and Bhatta, 2015). Tobacco use is socioculturally acceptable, especially among males in GCC countries (Maziak et al., 2014). Evidence indicates that youth who live in societies where tobacco use is socially acceptable are more susceptible to initiating tobacco use (Noland et al., 2016). Similarly, tobacco use among youth is associated with the status of tobacco use among parents, peers, and role models like teachers, physicians, and celebrities (US Department of Health and Human Services, 2012).

Beyond individual-level risk factors, the tobacco industry is another major factor to consider when discussing youth tobacco use. Youth ensure the tobacco industry’s continued profits. Tobacco industry documents have revealed many activities aimed at engaging young people in tobacco use behavior (Ling and Glantz, 2002). One of the tobacco industry's tactics is using direct or indirect marketing activities to promote tobacco use, such as advertisement, free distribution of cigarettes, sport sponsorships, and musical events. The tobacco industry links tobacco advertisements with social and entertainment events to influence youth who are more sensitive to ads than adults (Lapierre et al., 2017). The tobacco industry has also used the film industry to target youthful audiences through product placement and glamorizing the celebrity use of tobacco (Mekemson and Glantz, 2002).

### 1.2 Framework Convention on tobacco control (FCTC)

In 2003, the World Health Organization (WHO) developed the Framework Convention on Tobacco Control to help nations address tobacco use. The FCTC consists of evidence-based strategies to reduce both the demand for and supply of tobacco through changes to individuals' behaviors and physical environments (Table. 1) (United Nations, 2020). The effectiveness of the FCTC is well documented across countries (Chung-Hall et al., 2018). For example, smoke-free policies have an impact in reducing tobacco use, increasing cessation attempts, and creating norms against tobacco use (Hopkins et al., 2010, Alesci et al., 2003). Similarly, implementing a combination of several public education programs is associated with rendering tobacco use socially unacceptable and improving knowledge about the harmful effect of tobacco (Rennen et al., 2014, Wakefield and Chaloupka, 2000). Banning tobacco advertisement, promotion, and sponsorship (TAPS) is another proven approach to change the physical environment (Sargent et al., 2012, Shmueli et al., 2010, Blecher, 2008, Saffer and Chaloupka, 2000). To address tobacco supply among youth, FCTC prohibits the sale of tobacco products to minors. This ban impacts tobacco consumption among heavy-smoking youth who rely on commercial sources and changes youth perceptions about the ready accessibility of tobacco products (DiFranza, 2012, Nuyts et al., 2018).

**Table 1:** Key Framework Convention on Tobacco Control Strategies.

**Strategies Related to the Reduction of Demand for Tobacco**
• Price and tax measures to reduce the demand for tobacco
• Protection from exposure to tobacco smoke
• Regulation of the contents of tobacco products
• Regulation of tobacco product disclosures
• Packaging and labeling of tobacco products
• Education and public awareness
• Tobacco advertisement, promotion, and sponsorship
• Demand reduction measures concerning tobacco dependence and cessation
**Strategies Related to the Reduction of the Supply of Tobacco**
• Illicit trade in tobacco products
• Sales to and by minors
• Provision of support for economically viable alternative activities

Source: World Health Organization (World Health Organization, 2019).

In response to the issue of tobacco use, GCC countries ratified the FCTC treaty in 2006. Although the implementation of FCTC provisions leads to promising outcomes in many countries (Chung-Hall et al., 2018), knowledge about the relationship between the FCTC provisions and the susceptibility to initiating tobacco use is limited in GCC countries. Understanding whether FCTC provisions impact tobacco use among youth would help prioritize resources to specific provisions, improve the implementation process, and tailor tobacco control strategies to accommodate the cultural backgrounds of GCC countries. This study aimed to examine how susceptibility to initiate tobacco use changed over time in GCC countries and assessed the relationship between FCTC provisions and susceptibility to initiation of tobacco use among youth in GCC countries.

## 2 Method

### 2.1 Data Source

The current study obtained data from the Global Youth Tobacco Survey (GYTS), which is a school-based cross-sectional survey that collects data across time from students aged 13–15 (World Health Organization, 2013). The GYTS is publicly open data accessed from the Global Tobacco Surveillance System (https://www.cdc.gov/tobacco/global/gtss/). The survey was self-reported, anonymous, and voluntary. It used multistage sampling techniques that selected schools based on the enrollment size. The survey collected data about tobacco use and information of key topics related to implementing FCTC provisions (Global Youth Tobacco Survey Collaborative Group, 2012). Exemption was granted by IRB – Temple University.

### 2.2 Selection criteria

This study was limited to five GCC countries because 1) they are high-income countries located in the Eastern Mediterranean region, 2) they ratified the FCTC treaty, and 3) they have had multiple rounds of national youth data collection, including at least one before the ratification of the FCTC treaty. For each GCC country, two years of survey rounds were selected. One survey is pre-FCTC while the other survey is post-FCTC. Saudi Arabia was excluded because the first round of data collection before FCTC ratification included males only. Students who had never tried or experimented with any tobacco products were included in the analysis from the earliest and most recent round of data collection for each country.

### 2.3 Measures

The outcome variable in this study was *susceptibility to initiating tobacco use*. It was defined as the self-reported intention to initiate tobacco use and measured by the question: “at any time during the next 12 months, do you think you will use any form of tobacco?” Students who answered, “definitely not” were coded as “non-susceptible to initiate tobacco.” In contrast, all other students who answered, “probably not, probably yes, or definitely yes” were coded as “susceptible to initiate tobacco.” The outcome variable was recorded according to the validation study of Pierce et al. (1996). A dichotomous variable was created to represent the FCTC ratification indicator (0 = Pre-FCTC ratification, 1 = Post-FCTC ratification). Students who participated in the surveys collected pre-FCTC ratification were coded as 0 while those who took the survey after the FCTC ratification were coded as 1. Independent variables could be categorized into three domains: a) change over time, from pre- to post-FCTC ratification (FCTC ratification indicator); b) personal characteristics, including demographic, interpersonal, and social factors; c) FCTC provisions, including “protect from exposure to tobacco smoke,” “offer education and public awareness,” “ban TAPS,” and “prohibit the sale of tobacco to minors.” Table. 2 provides a codebook for selected variables.

**Table 2:** Codebook for Global Youth Tobacco Surveys, 2001–2018.

Variable	Recode response option	Bahrain Survey years: 2002, 2015	Kuwait Survey years: 2001, 2016	Oman Survey years: 2002, 2016	Qatar Survey years: 2004, 2018	UAE Survey years: 2002, 2013	Total
Susceptibility to initiate tobacco use Q. At any time during the next 12 months do you think you will use any form of tobacco?*	0 = Non-susceptible (Definitely not)	87.90%	88.10%	94.00%	91.00%	92.60%	91.90%
	1 = Susceptible (Probably not, Probably yes, & Definitely yes)	12.10%	11.90%	6.00%	9.00%	7.40%	8.10%
Time “change over time”Q. When did respondents answer the survey?	0 = Pre-FCTC ratification	47.00%	31.60%	42.70%	43.20%	43.50%	41.90%
	1 = Post-FCTC ratification	53.00%	68.40%	57.30%	56.80%	56.50%	58.10%
Age Q. How old are you?**	2 = 13 years old	34.70%	32.60%	14.00%	33.30%	34.80%	27.60%
	3 = 14 years old	33.90%	35.50%	41.20%	41.70%	36.90%	38.10%
	4 = 15 years old	31.40%	31.90%	44.70%	25.00%	28.30%	34.30%
Sex Q. What is your sex?	0 = Male	39.40%	38.50%	36.80%	40.40%	42.90%	39.80%
	1 = Female	60.60%	61.50%	63.20%	59.60%	57.10%	60.20%
Knowledge of harmful effects of SHSQ. Do you think the smoke from other people's tobacco smoking is harmful to you?*	0 = No (Definitely not)	29.50%	27.30%	32.50%	35.40%	35.50%	32.70%
	1 = Yes (Probably not, Probably yes, & Definitely yes)	70.50%	72.70%	67.50%	64.60%	64.50%	67.30%
Favorable belief about people who smoke at social gatherings Q. Do you think smoking tobacco helps people feel more comfortable or less comfortable at social gatherings?*	0 = Less comfortable or no difference	83.70%	86.60%	86.00%	85.60%	82.90%	84.70%
	1 = More comfortable	16.30%	13.40%	14.00%	14.40%	17.10%	15.30%
Parents’ smoking status Q. Do your parents smoke tobacco?	0 = None or do not know	79.50%	71.50%	89.50%	78.30%	80.60%	82.00%
	1 = At least one parent smokes (Both, Father only, & Mother only)	20.50%	28.50%	10.50%	21.70%	19.40%	18.00%
Friends’ smoking status Q. Do any of your closest friends smoke cigarettes?	0 = None	78.80%	80.00%	88.00%	84.60%	76.50%	81.30%
	1 = At least some friends smoke (Some of them, Most of them, & All of them)	21.20%	20.00%	12.00%	15.40%	23.50%	18.70%
Exposure to smoking in public places Q. During the past 7 days, on how many days has anyone smoked in your presence, inside any enclosed and/or outdoor public place, other than your home?***	0 = No (0 day)	53.10%	44.60%	65.50%	56.70%	59.00%	58.30%
	1 = Yes (1 to 2 days, 3 to 4 days, 5 to 6 days, & 7 days)	46.90%	55.40%	34.50%	43.30%	41.00%	41.70%
Exposure to anti-tobacco messages in media Q. During the past 30 days, did you see or hear any anti-tobacco media messages on television, radio, internet, billboards, posters, newspapers, magazines, or movies?	0 = No	35.50%	36.00%	32.60%	45.40%	35.70%	35.20%
	1 = Yes	64.50%	64.00%	67.40%	54.60%	64.30%	64.80%
Receive education about the harmful effect of tobacco at school Q. During the past 12 months, were you taught in any of your classes about the dangers of tobacco use?	0 = No (No & Not sure)	55.90%	65.60%	51.50%	67.40%	59.30%	57.70%
	1 = Yes (Yes)	44.10%	34.40%	48.50%	32.60%	40.70%	42.30%
Exposure to tobacco marketing in media Q. During the past 30 days, did you see any people using tobacco on TV, in videos, or in movies?	0 = No (I did not watch TV, videos, or movies in the past 30 days & No)	24.60%	26.40%	30.40%	31.00%	27.60%	28.30%
	1 = Yes	75.40%	73.60%	69.60%	69.00%	72.40%	71.70%
Exposure to tobacco promotion Q. Do you have something (for example, t-shirt, pen, backpack) with a tobacco product brand logo on it?*	0 = No	88.50%	91.90%	95.30%	94.00%	94.00%	93.60%
	1 = Yes	11.50%	8.10%	4.70%	6.00%	6.00%	6.40%
Exposure to tobacco sponsorship Q. Has a person working for a tobacco company ever offered you a free tobacco product?*	0 = No	95.80%	93.80%	95.40%	96.80%	95.40%	95.30%
	1 = Yes	4.20%	6.20%	4.60%	3.20%	4.60%	4.70%
Refuse to sell cigarettes due to legal age Q. During the past 30 days, did anyone refuse to sell you cigarettes because of your age?	0 = Not refused	0.60%	1.10%	0.50%	0.60%	1.00%	0.80%
	1 = Yes refused	0.80%	0.30%	1.80%	0.90%	0.40%	0.90%
	2 = Did not try to buy	98.70%	98.70%	97.80%	98.50%	98.60%	98.30%

(*) Surveys collected in GCC countries Pre-FCTC Ratification asked about smoking cigarettes while surveys conducted Post-FCTC Ratification asked for any form of tobacco; (**) Only those whose age between 13 and 15 years old included in the analysis; (***) Surveys collected in GCC countries Pre-FCTC Ratification asked about exposure to smoking “in places other than home” while surveys conducted Post-FCTC Ratification asked exposure to smoking in “enclosed” and “outdoor” public places in two questions. If a participant answered ≥ 1 day of exposure to smoking in either one of the questions, the response was coded as yes; (****) Responses were<15% in all countries.

### 2.4 Statistical analysis

**Fig. 1:**
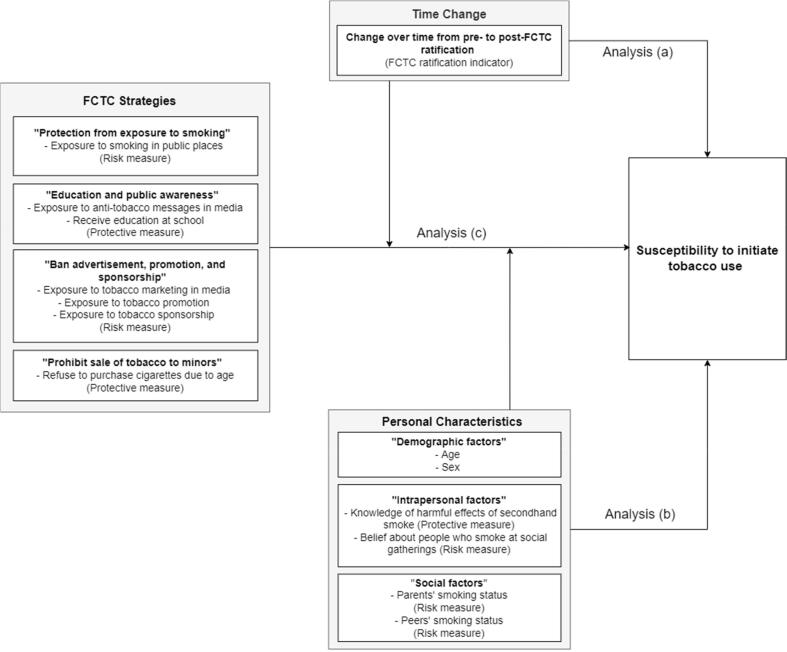
Conceptual Model Leading the Study Analysis.

Descriptive and bivariate analyses were conducted to understand the distribution of the data and relationships between variables. Complex samples statistics were conducted to provide valid inferences for populations measured in complex sample data (IBM Software, 2012). Three complex sample logistic regression analyses were performed to assess: a) the change of susceptibility to initiating tobacco use over time in relation to the FCTC ratification, b) the relationship between susceptibility and personal characteristics, and c) the association between susceptibility and FCTC provisions, controlling for confounding factors including FCTC ratification indicators and personal characteristics (Fig. 1). All statistics and standard errors were weighted to account for the complex sampling used in the GYTS. The analyses were performed using IBM SPSS Statistics 27 software (Corp, 2020).

## 3 Results

The overall response rates of surveys collected across GCC countries before the FCTC ratification ranged from 84.5% in Qatar to 96.9% in Oman, while the overall response rates of surveys conducted after the ratification of FCTC ranged from 76.9% in Bahrain to 93.2% in UAE. Details about response rates are found elsewhere (Centers for Disease Control and Prevention, 2021). The study sample included 349,878 adolescents in five GCC countries who had never tried or experimented with tobacco (females: 60.2%, males: 39.8%). Twenty-seven percent of adolescents were 13 years old, 38.1% were 14 years old, and 34.3% were 15. Approximately 8.2% of adolescents were susceptible to initiating tobacco use, of whom over 50% were female. Characteristics of participants who had never used tobacco were reported in Table. 3.

**Table 3:** Characteristics of Youth who had Never Smoke in Five GCC Countries by Susceptibility Status, 2001–2018^(^*^) (^**^)^.

		Bahrain			Kuwait			Oman			Qatar			UAE		
		Survey years: 2002, 2015			Survey years: 2001, 2016			Survey years: 2002, 2016			Survey years: 2004, 2018			Survey years: 2002, 2013		
Characteristics		Non-susceptible	Susceptible	p-value	Non-susceptible	Susceptible	p-value	Non-susceptible	Susceptible	p-value	Non-susceptible	Susceptible	p-value	Non-susceptible	Susceptible	p-value
**Age (Year)**	**13**	10927.2	1421.3	0.529	14433.4	1708.0	0.417	15211.4	888.8	0.375	5934.9	518.4	0.127	42269.5	3138.1	0.583
		34.8%	33.1%		33.1%	28.9%		14.1%	12.9%		33.7%	29.6%		34.9%	32.4%	
	**14**	10753.2	1393.4		15353.3	2241.8		44839.7	2434.1		7220.2	856.8		44311.9	3907.0	
		34.3%	32.4%		35.2%	38.0%		41.6%	35.4%		41.0%	48.9%		36.6%	40.4%	
	**15**	9682.1	1482.4		13882.7	1951.4		47715.0	3560.7		4452.8	376.9		34377.3	2635.1	
		30.9%	34.5%		31.8%	33.1%		44.3%	51.7%		25.3%	21.5%		28.4%	27.2%	
**Gender**	**Male**	11945.0	1972.6	0.052	16764.6	2299.7	0.882	39060.1	2982.5	0.294	7049.3	755.7	0.563	50379.4	5150.1	0.006
		38.3%	46.7%		38.5%	39.1%		36.4%	43.3%		40.1%	43.3%		42.0%	53.4%	
	**Female**	19252.7	2250.0		26739.5	3577.8		68120.0	3901.1		10531.2	991.0		69462.4	4495.1	
		61.7%	53.3%		61.5%	60.9%		63.6%	56.7%		59.9%	56.7%		58.0%	46.6%	
**Knowledge about dangers of secondhand smoking**	**No**	8369.4	2063.4	<0.001	11074.0	2366.6	0.001	32535.8	4595.4	<0.001	5895.4	932.9	<0.001	41019.0	5028.0	<0.001
		26.9%	48.9%		25.5%	40.4%		30.3%	66.8%		33.6%	53.7%		34.2%	52.3%	
	**Yes**	22759.5	2159.1		32379.0	3486.9		74916.3	2288.2		11639.9	804.9		78994.5	4577.5	
		73.1%	51.1%		74.5%	59.6%		69.7%	33.2%		66.4%	46.3%		65.8%	47.7%	
**Belief about smoking**	**Less comfortable/no difference**	26439.7	3085.7	<0.001	37427.7	4511.8	<0.001	92766.7	4888.1	<0.001	15032.6	1329.3	0.004	99622.6	6843.4	<0.001
		85.3%	72.5%		87.7%	78.8%		87.0%	71.0%		86.5%	77.4%		83.7%	72.2%	
	**More comfortable**	4541.8	1171.2		5272.5	1210.7		13897.1	1995.4		2348.2	389.0		19399.5	2638.9	
		14.7%	27.5%		12.3%	21.2%		13.0%	29.0%		13.5%	22.6%		16.3%	27.8%	
**Parents smoking**	**None/don't know**	25051.7	3060.4	0.001	31164.3	4092.3	0.417	97034.4	5362.1	0.001	13878.0	1260.8	0.056	97990.0	7114.1	0.008
		80.4%	71.8%		71.7%	69.6%		90.2%	77.9%		79.0%	72.0%		81.2%	73.5%	
	**At least one smoke**	6095.4	1203.5		12297.3	1790.5		10501.1	1521.5		3692.9	491.2		22674.9	2566.1	
		19.6%	28.2%		28.3%	30.4%		9.8%	22.1%		21.0%	28.0%		18.8%	26.5%	
**Freinds smoking**	**None**	25558.6	2428.2	<0.001	35704.0	3786.8	<0.001	95959.7	4720.3	<0.001	6209.4	645.1	<0.001	94240.8	5079.8	<0.001
		81.8%	56.7%		82.1%	64.2%		89.3%	68.6%		86.4%	69.9%		78.3%	53.6%	
	**At least some smoke**	5689.2	1852.6		7799.9	2109.1		11540.0	2163.3		974.9	277.8		26106.2	4404.6	
		18.2%	43.3%		17.9%	35.8%		10.7%	31.4%		13.6%	30.1%		21.7%	46.4%	
**Exposure to smoking in public places**	**No**	17124.2	1773.4	0.001	19940.5	2020.9	<0.001	70958.6	3414.4	0.012	10193.4	748.3	<0.001	73365.9	3612.0	<0.001
		54.7%	41.6%		45.9%	34.6%		66.5%	50.6%		58.1%	43.0%		60.8%	37.3%	
	**Yes**	14175.3	2492.3		23468.2	3826.2		35793.3	3333.9		7361.4	991.5		47330.6	6068.2	
		45.3%	58.4%		54.1%	65.4%		33.5%	49.4%		41.9%	57.0%		39.2%	62.7%	
**Exposure to anti-tobacco message in media**	**No**	10917.5	1680.6	0.318	15218.3	2576.6	0.005	34236.7	3038.2	0.010	7886.3	849.8	0.322	42740.0	3469.3	0.897
		35.0%	39.3%		35.0%	44.2%		31.9%	44.9%		45.1%	49.4%		35.6%	36.0%	
	**Yes**	20240.3	2600.2		28292.0	3259.1		73039.8	3734.3		9583.1	871.0		77199.3	6158.4	
		65.0%	60.7%		65.0%	55.8%		68.1%	55.1%		54.9%	50.6%		64.4%	64.0%	
**Receive anti-tobacco education at school**	**No**	17196.3	2507.0	0.252	28456.4	3985.5	0.563	54983.1	3903.3	0.130	11699.5	1185.7	0.705	71421.8	5781.2	0.908
		55.5%	59.2%		65.4%	67.6%		51.1%	57.3%		67.2%	69.1%		59.3%	59.7%	
	**Yes**	13785.4	1726.2		15065.1	1910.3		52517.2	2905.4		5708.7	530.3		49115.1	3899.0	
		44.5%	40.8%		34.6%	32.4%		48.9%	42.7%		32.8%	30.9%		40.7%	40.3%	
**Exposure to tobacco in media**	**No**	7744.5	942.2	0.393	11564.7	1429.1	0.520	33051.1	1796.5	0.300	5524.2	457.6	0.256	34012.5	2037.9	0.016
		24.9%	22.2%		26.6%	24.3%		30.7%	26.1%		31.5%	26.2%		28.2%	21.1%	
	**Yes**	23398.0	3297.3		31877.0	4443.3		74521.1	5087.1		12020.0	1290.2		86670.6	7642.3	
		75.1%	77.8%		73.4%	75.7%		69.3%	73.9%		68.5%	73.8%		71.8%	78.9%	
**Exposure to tobacco promotion**	**No**	27682.5	2872.8	<0.001	39791.5	4859.6	<0.001	102015.2	6127.2	0.008	16273.8	1454.6	<0.001	111049.6	8310.7	0.001
		91.0%	70.4%		92.9%	84.0%		95.6%	90.5%		94.8%	86.8%		94.4%	88.6%	
	**Yes**	2721.2	1205.5		3021.6	926.5		4654.8	644.9		896.2	221.5		6562.5	1065.5	
		9.0%	29.6%		7.1%	16.0%		4.4%	9.5%		5.2%	13.2%		5.6%	11.4%	
**Exposure to tobacco sponsorship**	**No**	30111.4	3711.3	<0.001	41232.2	5011.5	<0.001	102940.4	5869.0	0.027	16829.1	1649.5	0.454	113662.4	8233.2	<0.001
		96.9%	87.6%		94.8%	86.5%		95.7%	90.5%		96.9%	95.6%		95.8%	90.2%	
	**Yes**	961.5	525.9		2270.6	783.5		4635.6	619.6		543.4	75.3		5011.7	898.3	
		3.1%	12.4%		5.2%	13.5%		4.3%	9.5%		3.1%	4.4%		4.2%	9.8%	
**Refuse to sell cigarettes due to age**	**Not refused**	160.2	29.0	0.766	254.8	266.7	<0.001	429.6	140.1	0.003	73.7	41.4	<0.001	1219.2	89.5	0.041
		0.5%	0.7%		0.6%	4.6%		0.4%	2.1%		0.4%	2.4%		1.0%	0.9%	
	**Yes refused**	226.2	46.7		66.6	65.1		1643.3	306.0		110.1	60.4		344.8	123.8	
		0.7%	1.1%		0.2%	1.1%		1.5%	4.5%		0.6%	3.5%		0.3%	1.3%	
	**Did not try to buy**	30909.3	4185.2		43193.7	5524.8		105531.2	6350.8		17352.5	1625.9		118894.1	9466.9	
		98.8%	98.2%		99.3%	94.3%		98.1%	93.4%		99.0%	94.1%		98.7%	97.8%	

(*) non-integer numbers present weighted frequencies; (**) Significance is based on the adjusted F and its degrees of freedom.

### 3.1 Susceptibility to initiate tobacco use

The overall susceptibility to initiating tobacco use across the five GCC countries slightly decreased from 8.4% to 8% (χ^2^ = 0.7, P = 0.6). Susceptibility decreased over time from the pre- to post-FCTC ratification in four GCC countries, except the UAE. It statistically decreased in Bahrain from 15.6% in 2002 to 8.9% 2015 (χ^2^ = 24, P < 0.001) and in Qatar from 11.3% in 2004 to 7.3% in 2018 (χ^2^ = 10.9, P < 0.001). On the other hand, susceptibility increased in the UAE from 4.9% in 2002 to 9.3% in 2013 (χ^2^ = 24.2, P < 0.001). The change in susceptibility over time remained significantly reduced in Bahrain (AOR = 2, 95% CI = [1.4–2.7]) while it increased in the UAE (AOR = 0.5, 95% CI = [0.4–0.8]), after controlling for personal characteristics (Table. 4).

**Table 4:** Change Over Time in the Susceptibility to Initiate Tobacco Use among Youth in Five GCC Countries, 2001–2018.

GCC	Pre-FCTC Ratification* (2001–2004)	Post-FCTC Ratification* (2013–2018)	Chi-Square	P-value**	AOR***	CI	
						Lower	Upper
**Bahrain** Survey years: 2002, 2015	2621.9	1675.2	24.0	<0.001	2.0	1.4	2.7
	15.6%	8.9%					
**Kuwait** Survey years: 2001, 2016	1964.7	3936.5	0.7	0.5	1.1	0.8	1.5
	12.5%	11.6%					
**Oman** Survey years: 2002, 2016	3946.6	2937.0	9.8	0.1	1.4	0.8	2.7
	8.1%	4.5%					
**Qatar** Survey years: 2004, 2018	943.4	808.7	10.9	<0.001	1.7	1.0	2.7
	11.3%	7.3%					
**UAE** Survey years: 2002, 2013	2799.8	6880.4	24.2	<0.001	0.5	0.4	0.8
	4.9%	9.3%					

(*) non-integer numbers present weighted frequencies. (**) Significance is based on the adjusted F and its degrees of freedom; (***) Multivariate analysis: reference = Post-FCTC Ratification; Controlling for personal characteristics e.g., age, gender, knowledge about harm, favorable belief about smoking, parents' smoking, and friends' smoking.

### 3.2 Personal characteristics

There was no association between susceptibility to initiating tobacco use and demographic factors such as age and gender. After adjusting for change over time, susceptibility was negatively associated with knowledge about the dangers of secondhand smoking in five GCC countries (Bahrain: AOR = 0.4, 95% CI = [0.3–0.5]; Kuwait: AOR = 0.5, 95% CI = [0.3–0.8]; Oman: AOR = 0.2, 95% CI = [0.1–0.4]; Qatar: AOR = 0.4, 95% CI = [0.3–0.6]; UAE: AOR = 0.5, 95% CI = [0.4–0.6]). Susceptibility was positively associated with favorable beliefs about tobacco use in social gatherings in all GCC countries (Bahrain: AOR = 2.1, 95% CI = [1.4–3.2]; Kuwait: AOR = 1.7, 95% CI = [1.2–2.4]; Oman: AOR = 2.3, 95% CI = [1.5–3.6]; Qatar: AOR = 2.0, 95% CI = [1.3–3.0]; UAE: AOR = 1.7, 95% CI = [1.4–2.1]). Friends’ smoking status significantly increased the odds of susceptibility in four GCC counties (Bahrain: AOR = 2.8, 95% CI = [2.1–3.8]; Kuwait: AOR = 2.6, 95% CI = [1.9–3.6]; Oman: AOR = 3.3, 95% CI = [1.7–6.2]; UAE: AOR = 3.0, 95% CI = [2.2–4.1]) (Table. 5).

**Table 5:** Association Between Personal Characteristics and Susceptibility to Initiate Tobacco Use among Youth in Five GCC Countries, 2001–2018

GCC	Age			Gender			Protective Measure			Risk Measure			Risk Measure			Risk Measure		
							Knowledge about dangers of secondhand smoking			Favorable belief about smoking in social gatherings			Parents’ smoking			Friends’ smoking		
	(Reference = 15 year old)*			(Reference = female)			(Reference = no)			(Reference = Less comfortable or no difference)			(Reference = none or do not know)			(Reference = none)		
	AOR	CI		AOR	CI		AOR	CI		AOR	CI		AOR	CI		AOR	CI	
		Lower	Upper		Lower	Upper		Lower	Upper		Lower	Upper		Lower	Upper		Lower	Upper
**Bahrain** Survey years: 2002, 2015	1.0	0.7	1.4	1.3	0.9	1.9	0.4	0.3	0.5	2.1	1.4	3.2	1.3	0.9	1.7	2.8	2.1	3.8
	1.0	0.7	1.4															
**Kuwait** Survey years: 2001, 2016	0.9	0.6	1.3	0.9	0.7	1.3	0.5	0.3	0.8	1.7	1.2	2.4	1.0	0.8	1.3	2.6	1.9	3.6
	1.1	0.7	1.7															
**Oman** Survey years: 2002, 2016	0.9	0.4	1.8	1.0	0.5	1.7	0.2	0.1	0.4	2.3	1.5	3.7	2.1	1.2	3.6	3.3	1.7	6.2
	0.9	0.5	1.4															
**Qatar** Survey years: 2004, 2018	1.1	0.7	1.7	1.3	0.8	2.0	0.4	0.3	0.6	2.0	1.3	3.0	1.4	0.9	2.2	–	–	–
	1.5	1.0	2.3															
**UAE** Survey years: 2002, 2013	1.2	0.8	1.8	1.1	0.8	1.6	0.5	0.4	0.6	1.7	1.4	2.1	1.5	1.1	2.2	3.0	2.2	4.1
	1.2	0.8	1.9															

Note: Models were adjusted for change over time.

(*):AORs represent age 13 and 14, respectively.

### 3.3 Provisions of the Framework Convention on tobacco control

The odds of susceptibility to initiating tobacco use significantly increased with exposure to smoking in public places in Bahrain (AOR = 1.6, 95% CI = [1.2–2.2]), Kuwait (AOR = 1.6, 95% CI = [1.2–2.0]), Qatar (AOR = 1.9, 95% CI = [1.4–2.6]), and the UAE (AOR = 2.1, 95% CI = [1.6–2.7]). Examining the relationship between susceptibility and tobacco marketing activities revealed that the odds significantly increased if paired with tobacco promotion and sponsorship, as seen in Bahrain (AOR = 2.8, 95% CI = [1.9–4.2] and AOR = 3.2, 95% CI = [1.8–5.4]) and Kuwait (AOR = 2.2, 95% CI = [1.5–3.1] and AOR = 2.2, 95% CI = [1.5–3.3]). In the UAE, susceptibility increased with exposure to tobacco imagery through media (AOR = 1.7, 95% CI = [1.2–2.3]) and tobacco sponsorship (AOR = 2.0, 95% CI = [1.1–3.5]). While there was no association between susceptibility and anti-tobacco education at school in the GCC countries, exposure to anti-tobacco messaging in the media significantly decreased susceptibility in Kuwait (AOR = 0.7, 95% CI = [0.5–0.9]) and Oman (AOR = 0.6, 95% CI = [0.4–0.9]). Finally, susceptibility odds were negatively associated with refusal to sell tobacco in Kuwait (AOR = 0.1, 95% CI = [0.0–0.5]) and Qatar (AOR = 0.1, 95% CI = [0.0–0.7]) (Table. 6).

**Table 6:** Association Between FCTC Indicators and Susceptibility to Initiate Tobacco Use among Youth in Five GCC Countries, 2001–2018.

**GCC**	**Risk Measure**			**Protective Measure**			**Protective Measure**			**Risk Measure**			**Risk Measure**			**Risk Measure**			**Protective Measure**		
	Exposure to smoking in public places			Exposure to anti-tobacco message in media			Receive anti-tobacco education at school			Exposure to tobacco marketing in media			Exposure to tobacco promotion			Exposure to tobacco sponsorship			Refuse to sell cigarettes due to age		
	(Reference = no)			(Reference = no)			(Reference = no)			(Reference = no)			(Reference = no)			(Reference = no)			(Reference = not refused)*		
	AOR	CI		AOR	CI		AOR	CI		AOR	CI		AOR	CI		AOR	CI		AOR	CI	
		Lower	Upper		Lower	Upper		Lower	Upper		Lower	Upper		Lower	Upper		Lower	Upper		Lower	Upper
**Bahrain** Survey years: 2002, 2015	1.6	1.2	2.2	0.7	0.5	1.1	0.8	0.6	1.1	0.7	0.5	1.1	2.8	1.9	4.2	3.2	1.8	5.4	0.6	0.1	3.3
																			0.8	0.1	6.7
**Kuwait** Survey years: 2001, 2016	1.6	1.2	2.0	0.7	0.5	0.9	0.9	0.7	1.3	1.2	0.8	1.7	2.2	1.5	3.1	2.2	1.5	3.3	0.1	0.0	0.5
																			0.7	0.2	2.9
**Oman** Survey years: 2002, 2016	1.5	0.9	2.5	0.6	0.4	0.9	0.9	0.6	1.3	1.1	0.7	1.7	1.6	0.9	2.8	1.8	0.7	4.6	0.3	0.1	1.2
																			0.8	0.2	4.1
**Qatar** Survey years: 2004, 2018	1.9	1.4	2.6	0.8	0.6	1.1	1.1	0.7	1.9	1.2	0.8	1.8	2.4	1.4	4.2	1.0	0.4	2.7	0.1	0.0	0.7
																			0.9	0.1	8.2
**UAE** Survey years: 2002, 2013	2.1	1.6	2.7	1.1	0.9	1.5	1.0	0.8	1.4	1.7	1.2	2.3	1.5	0.9	2.6	2.0	1.1	3.5	1.0	0.2	4.7
																			4.1	0.4	40.9

Note: Models were adjusted for change over time and personal characteristics including demographic factors: age and gender, intrapersonal factors: knowledge about dangers of secondhand smoking and favorable belief about smoking, and social factors: parents’ and friends’ smoking status.

(*): AORs represent yes refused and did not try to buy, respectively.

## 4 Discussion

This study examined change in susceptibility in the GCC region and assessed youth’s self-reported susceptibility to initiating tobacco use to help in identifying at-risk adolescents, thereby providing a pathway for tobacco control interventions further upstream in the tobacco use process. Susceptibility to initiating tobacco use has decreased in most GCC countries since the FCTC ratification. The study provided initial evidence of factors associated with susceptibility to use tobacco that may be unique to youth in the GCC, as compared to other regions. It revealed that FCTC provisions contribute to preventing the initiation of tobacco use among youth in GCC countries.

Compared to countries in the Eastern Mediterranean and Southeast Asia (Veeranki et al., 2014, Guindon et al., 2008), youth in the GCC countries reported a lower susceptibility to initiating tobacco use. At least two GCC countries (Bahrain and Qatar) showed a statistical reduction in the percentage of students who were willing to initiate tobacco use. Yet, the percentage of students who had never used tobacco and intended to initiate tobacco use increased in the UAE. The increasing trend of the diverse populations that include expatriate employees in the UAE may explain our findings (Ali et al., 2020, Telecommunications and Digital Government Regulatory Authority, 2020). As of 2015, the percentage of expatriate workers in the UAE reached 88.5%, which is the highest across all GCC countries (Gulf Research Center, 2015). Tobacco use among non-UAE residents is higher than among UAE nationals (31% vs. 24%) (Ali et al., 2020, Al-Houqani et al., 2018). Also, the Middle East Tobacco Association located in Jabil Ali in the UAE has historically engaged in illegal tobacco trade activities including influencing tobacco marketing and weakening tobacco control activities in the UAE (Holden, 2017).

Several studies indicate that tobacco use was higher among males than females in Eastern Mediterranean countries (Khattab et al., 2012, Usmanova and Mokdad, 2013). This study found that about 8.2% of the teens in GCC were susceptible to initiate tobacco use, of whom above 50% were females (Table. 3). This finding may indicate that the tobacco industry is targeting young females as a strategy to recruit new tobacco users (Usmanova and Mokdad, 2013, Hunt et al., 2020). It could also be attributed to the gender role and underreporting of tobacco use among females in GCC countries due to the social stigma associated with tobacco use in the Middle East (Maziak et al., 2014, Jarallah et al., 1999, Aslam et al., 2014). Females in GCC countries are less open to sharing their smoking status with others and may hide their smoking status during an interview or physician visits (Maziak et al., 2014, Jarallah et al., 1999); examining self-reported susceptibility identified those who were willing to initiating tobacco use. The cultural norm that stigmatizes tobacco use by females may lead to delivering unequal tobacco control information for females compared to males (Greaves and Organization, 2007). Thus, extended tobacco control efforts targeting both male and female youth are needed to prevent tobacco use and experimentation.

Consistent with the existing literature, this study shed light on the primary role of intrapersonal factors. Knowledge about the risks of tobacco use and its short and long-term consequences has been used as a protective intervention to prevent tobacco use, while a positive social image about tobacco use was determined as a risk factor for tobacco initiation (Kasim et al., 2016). In relationship to other studies conducted in several countries, youth who have never used tobacco are more influenced by their smoking friends than their smoking parents (Distefan et al., 1998, de Vries et al., 2003). In agreement with previous studies (Biddle et al., 1980, US, 2012), comparing and contrasting peer and parent influences showed that peers’ *behaviors* have a higher impact on youth, whereas parents’ *norms* have a higher effect on youth. Consequently, tobacco control programs should apply a social network approach and peer-led tobacco prevention strategies to reinforce negative normative attitudes toward tobacco use among youth (Mohammed et al., 2016, Seo and Huang, 2012). The study finding also suggests an intensive implementation of smoking cessation programs and smoke-free policies in frequently visited youth places.

The study identified a relationship between key FCTC provisions and youth susceptibility to tobacco use. Like results in several studies (Okoli and Kodet, 2015, Xi et al., 2016), exposure to smoking in public places was a significant factor for those who had never used tobacco in the GCC countries. Smoking in public places, such as restaurants and cafes, is allowed in designated areas in at least Bahrain, Saudi Arabia, and the UAE. It is also a common practice to gather in cafés and restaurants and share waterpipes (Maziak et al., 2014). In a collectivistic society like Arab society, where the culture appreciates conformity, the influence of friends who smoke increases the risk of tobacco use among individuals who have never used tobacco (Liu et al., 2017). This evidence, along with our findings, suggests the necessity of enforcing complete smoke-free policies in public places to protect youth from exposure to smoking in public places. Smoke-free policies should, however, accompany various interventions aimed at engaging and empowering youth to reframe their beliefs about tobacco use and avoid rebellion against policy (Sargent and DiFranza, 2003, Douglas et al., 2016).

Examining the relationship between susceptibility and anti-tobacco media messaging and school-based education suggests that anti-tobacco media campaigns may lead to better outcomes than school-based education, media campaigns may reach a larger population and meet youth where they are, rather than limiting engagement to the institution. Previous studies found that delivering information about tobacco risks in school settings, as a part of curriculums or lectures alone, has little impact on students’ behavior change in GCC countries (Al-Lawati et al., 2017, Al-Dahshan et al., 2019). Our study revealed that being exposed to anti-tobacco messaging in media is negatively associated with susceptibility in Kuwait and Oman. This finding may reflect variations in constructing and implementing anti-tobacco educational materials across GCC countries. Effective anti-tobacco educational materials should consider the duration/frequency of the exposure, context, and audience (Wakefield and Chaloupka, 2000, Duke et al., 2015, Farrelly et al., 2003, Farrelly et al., 2017). There is limited information to describe anti-tobacco educational programs across all GCC countries (Monshi and Ibrahim, 2021). As younger generations have become more liberal, anti-tobacco messages should be customized to shared characteristics of this age group in order to effectively influence their behaviors (Schmid et al., 2008, Harb, 2014). Future research may examine variations in anti-tobacco public education interventions conducted in GCC countries and study their effects on tobacco use.

Documents from the tobacco industry revealed the negative influence of the tobacco industry in GCC countries, including lobbying public figures and weakening the implementation of tobacco control policies (White and Hammond, 2001, Hammond and White, 2001). Tobacco marketing activities such as distributing free tobacco, promotion, and exposure to tobacco imagery in media were found in GCC countries. In agreement with other studies, the results of this study found that tobacco marketing impacts youth who never used tobacco (US Department of Health and Human Services, 2012, English et al., 2016). Multiple characteristics made GCC countries vulnerable to the tobacco industry, including the rapid urbanization post-oil discovery in the 1930 s. Living in urban areas has been associated with higher tobacco marketing activities (Savell et al., 2015, Farley et al., 2019). Also, GCC countries have relatively young populations (∼50% of the total population are under 25) (AlMunajjed and Sabbagh, 2011). These characteristics prompted the tobacco industry to aggressively market the new generation of tobacco products such as electronic cigarettes and heated tobacco products (White and Hammond, 2001, Hammond and White, 2001, Tobacco Tactics, 2020). With higher access to the internet and social media apps in GCC countries, youth may be at higher risk of tobacco marketing influences (Dubai Press Club, 2020, Collins et al., 2019). Ongoing surveillance is needed to assist policy makers in allocating resources toward more effective tobacco control policies and monitoring tobacco industry activities in the region. Upcoming research should explore tobacco industry activities in GCC countries and examine how industry interferes in each country in comparison to others.

### 4.1 Limitations

This study has several limitations. First, the study relied on cross-sectional surveys collected from students in school settings. In contrast to longitudinal data, cross-sectional surveys did not capture the effect of the policy due to the ambiguous temporal trends that occurred before the FCTC ratification. For instance, GCC countries may have implemented tobacco control policies way before the FCTC ratification, but cross-sectional surveys were not able to capture these efforts. Although a cross-sectional survey is insufficient to infer a causal relationship, it allows for the examination of the strength of the relationship between factors. Also, self-reported measures may be prone to recall and social desirability bias. Although the gross percentage of school enrollment increased in the GCC region (World Bank, 2020), there may be a bias, since the respondents may not represent the behaviors of youth who are not in school. Second, the two surveys (pre-FCTC & post-FCTC ratification) asked variable questions. For instance, most initial survey questions concentrated on cigarette use, while the post-FCTC survey comprehensively inquired about tobacco products. Since waterpipes are a common tobacco product in GCC countries, a limitation in asking the first survey question may lead to underestimating susceptibility to initiating tobacco use over time in GCC countries. Third, we used one question from the survey to measure susceptibility to initiate tobacco use. In contrast, several studies used multiple questions (Veeranki et al., 2014, Aslam et al., 2014, Veeranki et al., 2015), including “Do you think you will be using tobacco 5 years from now?” and “If one of your friends offered you a cigarette, would you smoke it?” The first question was not considered in this study because it was not asked consistently across the five GCC countries. We also found a high correlation between the second question and our question when we conducted a correlation analysis between the two variables. Therefore, we were satisfied with using one measure. Fourth, we acknowledge that policy adoption requires sufficient time to show outcomes. The unequal time difference between FCTC ratification and data collection in each GCC country may inaccurately estimate change over time in susceptibility to initiating tobacco use. Finally, this study did not consider tobacco control efforts made at the local level in each country, such as conducting anti-tobacco campaigns and enforcing tobacco control local ordinances. Limited information regarding local efforts hinders our ability to control for these confounding factors.

## 5 Conclusion

This study identified 8.2% of adolescents who were susceptible to initiating tobacco use in five GCC countries between 2001 and 2018. Detection of factors associated with the pre-experimentation stage of tobacco use can help explain the reason for the slow reduction of tobacco use in the GCC region. Personal factors and social context have a great impact on tobacco use. Yet, FCTC provisions may independently contribute to preventing the initiation of tobacco use by influencing both environment and behavior. Although the GCC countries share political, economic, and sociocultural characteristics, the impact of FCTC provisions differs across countries. This outcome opens a window of opportunity for policymakers in GCC countries to share lessons learned about tobacco control and allocate resources accordingly. The implementation of tobacco control at the population level may require tailoring tobacco control policies and coordination between agencies. Thus, the ongoing surveillance would help track tobacco use prevalence, monitor tobacco control progress, amend policies when unintended consequences occur, and allocate resources for more impactful tobacco control interventions.

## 6 Authorship and Originality

The authors declare that this manuscript has not been previously published and is not currently under consideration by any other journal. All authors approved the present submitted version and their institutions have no objections to the manuscript’s contents.

## Funding

The authors declare there is no specific fund for this research from any public, commercial, or not-for-profit agencies.

## CRediT authorship contribution statement

**Sarah S. Monshi:** Conceptualization, Formal analysis, Writing – review & editing. **Jingwei Wu:** Conceptualization, Formal analysis, Writing – review & editing. **Bradley N. Collins:** Conceptualization, Writing – review & editing. **Jennifer K. Ibrahim:** Conceptualization, Formal analysis, Writing – review & editing, Supervision.

## Declaration of Competing Interest

The authors declare that they have no known competing financial interests or personal relationships that could have appeared to influence the work reported in this paper.
